# Evaluation of the Efficacy of Magnesium Sulfate in Reducing Blood Loss in Functional Endoscopic Sinus Surgery: A Randomized Double-Blinded Controlled Trial

**DOI:** 10.7759/cureus.38636

**Published:** 2023-05-06

**Authors:** Anandpandi Preethi, Rajagopalan Venkatraman, Urkavalan Karthika, Aravindan Rangapriya

**Affiliations:** 1 Anaesthesiology, SRM Institute of Science and Technology, Chennai, IND

**Keywords:** magnesium sulphate, chronic, sinusitis, endoscopic sinus surgery (fess), blood loss

## Abstract

Objectives

The primary concern in functional endoscopic sinus surgery (FESS) is maintaining a clear and unobstructed surgical field. Achieving this objective necessitates controlled hypotension, which can aid in the surgical dissection process and reduce the overall duration of the operation. This study aims to evaluate the efficacy of a sole bolus injection of intravenous magnesium sulfate in FESS. The outcomes measured include blood loss, surgical field grading, the additional intraoperative requirement of fentanyl, stress attenuation during laryngoscopy and endotracheal intubation, and extubation time.

Methods

In this prospective, double-blinded, randomized control trial (CTRI/2021/04/033052), 50 patients scheduled for FESS were randomly divided into two groups: Group M received 50 mg/kg MgSO_4_ in 100 ml normal saline, and Group N received 100 ml plain normal saline 15 min before induction. The study assessed total blood loss, measured by blood collected from the surgical field and weighing gauze. The surgical field grading was assessed by a six-point Fromme and Boezaart scale. We also observed stress attenuation during laryngoscopy and endotracheal intubation, additional intraoperative fentanyl requirements, and time taken for extubation. The sample size was estimated using the G power calculator 3.1.9.2 (http://www.gpower.hhu.de/). Data were entered in Microsoft Excel (Microsoft Corporation, Redmond, WA) and analyzed using Statistical Package for Social Sciences version 20.0 (IBM Corp., Armonk, NY).

Results

The demographic data and duration of the surgery were comparable in both groups. The total blood loss in Group M was 100.40 ml ±60.71 ml, which is lower than Group N (133.80 ml ±59.7 ml) with a p-value of 0.016. In addition, the surgical field grading was also better in Group M. The total vecuronium consumption was significantly lower in Group M, which was (7.23±0.84 mg); in Group N, it was (10.64±1.74 mg) with a p-value of 0.0001, respectively. The dosage of additional fentanyl in Group N was 38.46 mcg ± 8.99 mcg, more than in Group M (33.64 mcg ± 11.20 mcg). The time required for extubation was similar in both groups. The duration of the surgery was significantly more significant in Group M (150.0 ±31.36) than in Group N (205.0 ±32.79), with a p-value of 0.0001, respectively.

Furthermore, the mean arterial pressure after induction, at 2 min and 4 min after laryngoscopy, was less in Group M, with p=0.001, p=0.003, and p<0.0001, respectively, when compared with Group N. The heart rate after induction, at 2 min and 4 min after laryngoscopy, was also less in Group M, with p=0.016, p=0.003, and p=0.003, respectively, when compared with Group N.

The Ramsay Sedation Score was higher in Group M than in Group N's fourth, eighth, and sixteenth hour, with p=0.001, p=0.021, and p=0.001, respectively, in the postoperative period. The sedation score was statistically insignificant after that. No complications were encountered during the study.

Conclusion

We conclude that a single bolus dose of MgSO_4_ reduced surgical blood loss more effectively than in the control group. The surgical field grading was also better in Group M, as was the stress attenuation during laryngoscopy and endotracheal intubation. The intraoperative fentanyl requirement was not statistically significant. The time for extubation was similar between the groups. No other adverse effects were encountered during the study.

## Introduction

Functional endoscopic sinus surgery (FESS) is a procedure used to treat chronic sinusitis, nasal polyps, mucoceles, foreign bodies, and recurrent epistaxis [1,2]. It targets the idea that the obstruction of the anterior ethmoid cells in the middle meatus area causes most paranasal sinus disorders. Less common indications of the need for FESS include recurrent acute sinusitis, symptomatic nasal polyps, mucoceles, foreign bodies, recurrent epistaxis, repair of CSF leaks, biopsy of nasal growths, orbital decompression, and pituitary surgery [3,4].

Bleeding during FESS can reduce visibility in the surgical area, making the procedure more difficult and increasing the risk of complications. Therefore, it is essential to control bleeding during FESS to ensure a successful outcome [3]. Controlled hypotension is used during surgery to temporarily lower blood pressure to reduce blood flow and bleeding in the surgical area, which can create a relatively bloodless surgical field, making surgical dissection easier and improving visibility for the surgeon [1].

Controlled hypotension means administering medications that dilate the blood vessels and slow the heart rate, allowing the surgeon to work with a clearer view of the surgical area [1]. However, it is essential to maintain a safe level of blood pressure to avoid adverse effects on the patient’s health. Controlled hypotension can be achieved through the use of various medications. Some commonly used drugs include beta-blockers, nitroglycerine, sodium nitroprusside, and magnesium sulfate (MgSO_4_). It is important to note that the choice of medicine and dosage depends on the patient's medical history, current condition, and specific surgery requirements [5]. For example, MgSO_4_ is a calcium channel blocker. It has properties that depress the heart muscle while also dilating blood vessels, which can lead to a reduction in the surgery duration, arterial pressure, heart rate, and blood loss [6]. In addition, it prolongs the effect of depolarizing and non-depolarizing muscle relaxants. Magnesium has no direct antinociceptive effects; it inhibits calcium ions from entering cells by blocking N-methyl-D-aspartate (NMDA) receptors, resulting in an analgesic effect [7].

When giving magnesium sulfate, there are minimal side effects with standard therapeutic doses, but magnesium sulfate has a broad therapeutic index. Patients most commonly complain of minor facial flushing, nausea, vomiting, and warmth with the administration; however, symptoms typically resolve spontaneously. In patients with neuromuscular disease, such as myasthenia gravis, the neuromuscular function may worsen at lower medication concentrations. If given rapidly or in high doses, patients may experience transient hypotension due to smooth muscle inhibition causing a vasodilatory effect that will resolve [8].

This study aims to evaluate the efficacy of a single bolus dose of intravenous MgSO_4_ in reducing blood loss and improving the surgical field grading during FESS. The secondary objectives are to assess the additional intraoperative requirements of fentanyl, stress attenuation during laryngoscopy and endotracheal intubation, and time for extubation.

## Materials and methods

The study was conducted from May 1, 2021, to October 31, 2022, and was a prospective, double-blinded, randomized controlled trial. After obtaining the institutional ethics committee approval (IEC NO 2394), the study was registered in Clinical Trial Registry-India (CTRI/2021/04/033052). Patients were included in the study if they were 18-60 years old, had American Society of Anesthesiologists (ASA) physical status I and II, had body mass index ranging from 18.5 to 24.99, and had serum magnesium between 1.7 and 2.4 mg/dL. Exclusion criteria included the patients on beta blockers, patients undergoing FESS for sinonasal tumors, patients with coagulation disorders, patient refusal, and allergy to the study drug.

The randomization was done by block method, using a computer-generated sequence and opaque sealed envelopes, with the support staff of the institute generating the arrangement and the principal investigator allocating patients to the groups. After opening the envelope, an anaesthesiologist prepared a 100 ml solution of either MgSO_4_ or NS, according to the group allotted. After that, he took no further part in the study. Another anaesthesiologist, blinded to the groups, administered anesthesia and monitored the patients intraoperatively. The surgeon was also blinded to the treatment allocation. After obtaining informed consent from 50 eligible patients, they were randomly assigned to two groups: Group M received MgSO_4_ in a bolus dose of 50 mg/kg in a volume of 100 ml, which was derived from the study by Hamed et al. [9]. Group N was the control group that received normal saline (NS) 100 ml.

Before the surgery, patients were given premedication, including tablets of alprazolam, ranitidine, and metoclopramide, on the night before and the morning of the surgery, along with sips of water. Various monitors were attached to the patients in the operating room intraoperatively, including a pulse oximeter, non-invasive blood pressure cuff, electrocardiogram, capnography, and neuromuscular monitoring. All the patients received 100 ml of the solution as prepared by the first anesthesiologist 15 min before induction. Then, the patients were given general anesthesia, which included fentanyl (2 mcg/kg), propofol (2 mg/kg), and vecuronium (0.1 mg/kg) intravenously (iv), followed by intubation using sevoflurane and a combination of 50% O_2_ and 50% N_2_O to maintain the desired level of anesthesia. 

Heart rate and mean arterial pressure (MAP) were monitored continuously and recorded at different time points: baseline, before and after induction, every 2 min for 10 min after laryngoscopy, every 10 min for 1 hour, and every 15 min until the end of the surgery. Fentanyl was administered as a bolus if heart rate or systolic blood pressure increased by more than 20%. The number of patients who required additional doses of fentanyl was recorded for both groups. To maintain the desired level of muscle relaxation during the procedure, vecuronium boluses were repeated as necessary to keep a train of four (TOF) count of less than 0.3.

After the surgery, the anesthetic gases were gradually reduced and stopped, and patients were given 100% oxygen for support during ventilation. Patients received intravenous neostigmine and glycopyrrolate to reverse the muscle relaxant effect after achieving a TOF >0.7. The patients were extubated after completing recovery criteria and achieving a TOF >0.9. The time taken from finishing the surgical dressing to removing the endotracheal tube was recorded as the time for extubation.

The total blood loss was measured by measuring the blood collected from the surgical field and weighing gauze pieces. Each gauze piece was considered on a digital weighing machine and recorded, with 1 g of weight equivalent to 1 ml of blood. The surgeon assessed the surgical field grading, which was done using a six-point Fromme-Boezaart scale, a standardized scale for measuring the amount of blood loss in a surgical setting [10]. After the surgery, patients' hemodynamics were monitored continuously and recorded every 4 to 24 hours. In addition, hemoglobin and packed cell volume were rechecked six hours after the surgery. Moreover, the patient's level of sedation was evaluated using the Ramsay Sedation Score (RSS) every 4 hours for up to 24 hours postoperatively [11]. We looked for postoperative nausea, vomiting, increased sedation, and residual neuromuscular deficit.

The sample size was estimated using the G power 3.1.9.2 calculator program from the University of Kiel, Germany (http://www.gpower.hhu.de/). A pilot study was conducted with 10 patients, which provided mean blood loss values for both groups. The blood loss in the MgSO_4_ group was 230.45 ml ± 45.04 ml, and in the NS group was 177.34 ml ± 56.65 ml. Considering a 95% significance level and 95% power, we calculated the sample size to be 44. However, to account for potential dropouts, the total sample size was 50. The patients from the pilot study were not included in the final analysis. The data were analyzed and presented as mean, standard deviation, frequency, and percentage. For normalcy of distribution, the Skewness-Kurtosis All test was used. Continuous variables were compared between the groups using an independent sample t-test. Comparing categorical variables between the groups was created using the Pearson chi-square test. A p-value of less than 0.05 using a two-tailed test was considered statistically significant.

## Results

**Figure 1:**
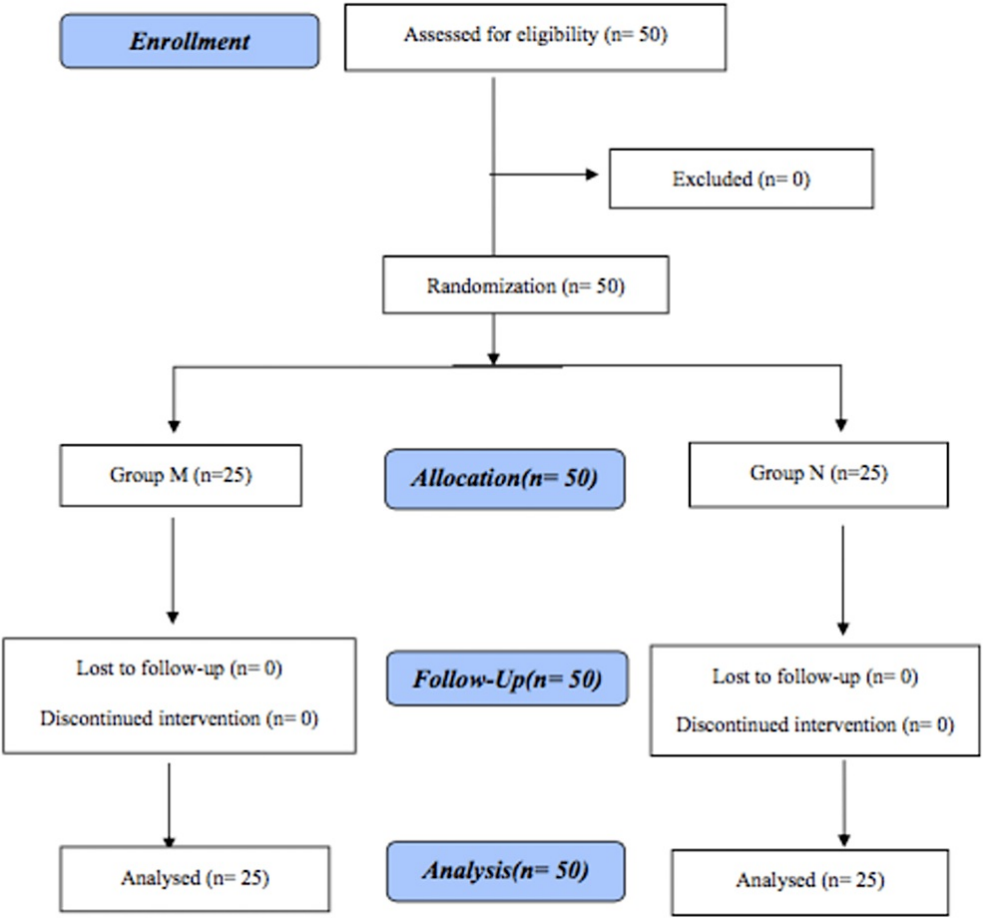
CONSORT flow chart CONSORT: Consolidated Standards of Reporting Trials

Figure 1 shows the Consolidated Standards of Reporting Trials (CONSORT) diagram.

The CONSORT flow chart illustrates the flow of patients throughout the study. It shows that all patients randomly assigned to a treatment group completed the study; there were no dropouts or withdrawals. The two groups were comparable in demographic profile, preoperative hemoglobin, packed cell volume, and preoperative serum magnesium level. However, the duration of the surgery was significantly more significant in Group M (150.0 ±31.36) than in Group N (205.0 ±32.79), with a p-value of 0.0001. The results are summarized in Table 1.

**Table 1:** Demographic characteristics Values are mean ± standard deviation (SD) or number of patients; *p-value is not statistically significant; ^†^p-value is statistically significant

Parameters	Group M	Group N	P-value
Age (years)	32.92 ± 11.03	35.56 ± 10.73	0.395^*^
Weight (kg)	69.64 ± 15.42	63.24 ± 12.87	0.118*
Preoperative Hb (g/dl)	13.08 ± 2.05	13.16 ± 1.85	0.891*
Postoperative Hb (g/dl)	12.86 ± 1.87	13.33 ± 1.60	0.342*
Preoperative PCV	40.72 ± 3.93	41.28 ± 3.08	0.577*
Postoperative PCV	39.92 ± 3.48	41.04 ± 3.10	0.235*
S.Magnesium (mg/dl)	1.80 ± 0.17	1.70 ± 0.21	0.106*
Duration of surgery (min)	150.0±31.36	205.0±32.79	0.0001^†^

The blood loss for Group M was 100.40 ml ± 60.71 ml and for Group N, 133.80 ml ± 59.70 ml. This study found a statistically significant difference in total blood loss between groups M and N, as indicated by the p-value of 0.016. When the surgical field grading was compared between the groups, it was found that 56% of the patients in Group M were in Grade 1, 44% were in Grade 2, and no patient was in Grade 3 or 4. In contrast, 24% of the patients in Group N were in Grade 1, 36% were in Grade 2, 32% were in Grade 3, and 8% were in Grade 4. The data showed a statistically significant difference in surgical field grading between the two groups, with a p-value of 0.004.

**Figure 2:**
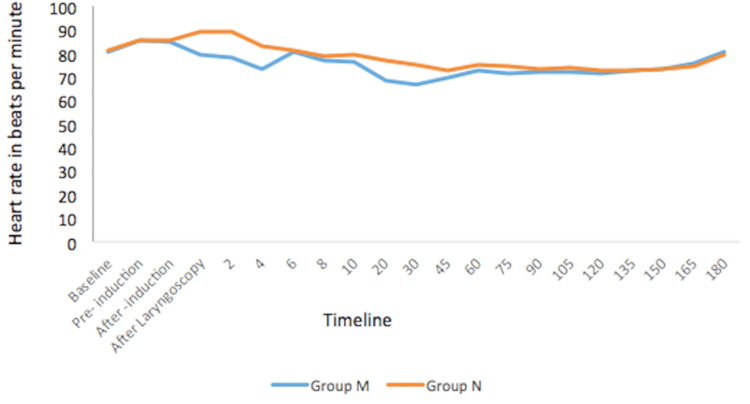
Changes in the heart rate

There was no statistically significant difference in the heart rate between the groups at baseline and after induction. However, there was a considerable difference in the heart rate after laryngoscopy, at 2 min and 4 min, with p-values of 0.016, 0.003, and 0.003, respectively. The heart rates between the groups at 6 to 180 min were not significantly different (Figure 2).

Additionally, there was no significant difference in postoperative heart rate between the groups for up to 24 hours postoperatively.

**Figure 3:**
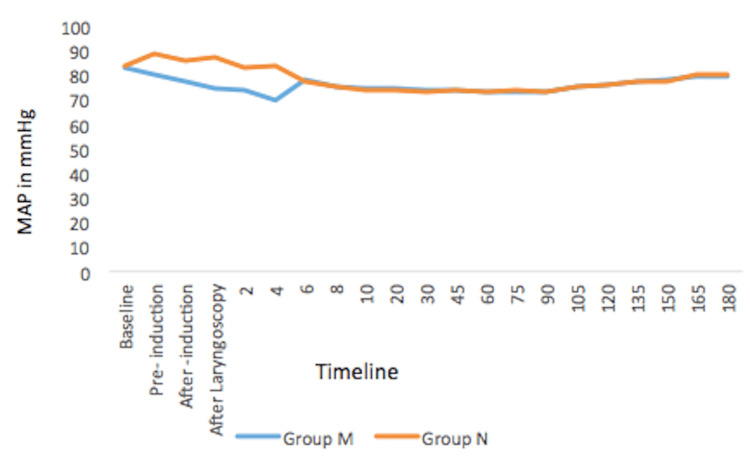
Changes in the mean arterial pressure (MAP)

There was no statistically significant difference in MAP between the groups at baseline. However, there was a significant difference in MAP between the groups at pre-induction (p<0.0001), after induction (p<0.0001), after laryngoscopy (p=0.001), at 2 min (p=0.003), and 4 min (p<0.0001) following laryngoscopy (Figure 3).

On the other hand, there was no significant difference in the postoperative MAP between the groups.

There was a statistically significant difference in RSS between the groups at 4 hours, 8 hours, and 16 hours, with p-values of 0.001, 0.021, and 0.001, respectively, and Group M reported more sedation. However, there was no significant difference in RSS between the groups at 12 hours, 20 hours, and 24 hours (Table 2).

**Table 2:** Comparison of Ramsay Sedation Scores values are in median; ^†^p-value is statistically significant; *p-value is not statistically significant

Hours	RSS	Group M	Group N	p-value
4	1	5	0	0.001^†^
	2	14	25	
	3	6	0	
8	1	6	13	0.021^†^
	2	14	12	
	3	5	0	
12	1	19	22	0.269*
	2	6	3	
16	1	24	25	0.001^†^
	2	1	0	
20	1	25	25	n/a
24	1	25	25	n/a

The total vecuronium consumption was significantly reduced in Group M, which was 7.23 ± 0.84 mg; in Group N, it was 10.64 ± 1.74 mg with a p-value of 0.0001, respectively. The additional fentanyl requirement in Group M was 33.64 mcg ± 11.20 mcg; in Group N, it was 38.46 mcg ± 8.99 mcg. No statistically significant difference was found in the additional fentanyl required between the two groups, with a p-value of 0.254. Moreover, no significant difference was in the time taken for extubation between the groups, with 10.40 min ± 2.86 min for Group M and 10.36 min ± 2.80 min for Group N (p=0.960).

There was no incidence of complications like nausea, vomiting, increased sedation, and residual neuromuscular deficit reported during the study.

## Discussion

Controlled hypotension plays a significant role in FESS in providing a clear surgical field and reducing the surgery duration. Our study results show that a single bolus dose of intravenous MgSO_4_ effectively reduces intraoperative blood loss, improving the visibility of the surgical area. The amount also influenced stress attenuation during laryngoscopy and endotracheal intubation without prolonging the time for extubation and any other adverse effects.

This study showed a statistically significant difference in total blood loss between Group M and Group N, with a p-value of 0.016. This finding is consistent with the study by Liu et al., who also compared the use of MgSO_4_ with placebo in patients undergoing endoscopic sinus surgery. They concluded that MgSO_4_ was associated with a significantly lower amount of intraoperative blood loss and improved the surgical field grading as compared to a placebo [12].

A study by Ajisha Aravindan et al. compared the use of MgSO_4_ or diltiazem and a placebo group in patients undergoing FESS. It concluded that adding MgSO_4_ resulted in significant blood loss reduction and improved surgical field grading [13].

In our study, we used the Formme-Boezaart surgical field grading to assess the area of surgery [10]. We observed that in the group that received MgSO_4_, most cases (n=14) had a surgical field grading of 1, followed by surgical grading of 2 (n=11). In the placebo group, six patients had grade 1, nine had grade 2, eight had grade 3, and two had a grade 4 surgical field. No patients had a field grade of 0. This difference in surgical field grading was statistically significant.

In our study, we have tried to understand the effect of using MgSO_4_ before intubation and its ability to attenuate this stress response; we gave MgSO_4_ in the dosage of 50 mg/kg 15 min before induction. The heart rate and MAP were significantly reduced after 2 min and 4 min laryngoscopy but insignificant in Group M. Kiran Jangra et al. conducted a study in which there was a considerable difference in the heart rate and MAP, which was better in the MgSO_4_ group than the esmolol and the control groups [14]. A study conducted by Elsharnouby NM et al. concluded that MgSO_4_ reduces MAP, heart rate, and blood loss [15]. In addition, the total consumption of vecuronium decreased significantly in Group M.

In our study, additional 0.5 mcg/kg fentanyl was given if the heart rate and systolic blood pressure increased more than 20%, was comparable in both groups, and was found to be statistically insignificant. Liu et al. conducted a study in which there was no significant reduction in the requirement for fentanyl, which is similar to our study [12].

The time taken for extubation was similar in both groups and statistically insignificant. Furthermore, a study conducted by Bayram A et al. comparing MgSO_4_ and dexmedetomidine concluded that MgSO_4_ does not prolong the time for extubation, which is analogous to our study [16]. 

Our study had a few limitations. First, we used only the bolus dose of MgSO_4_ and did not use the infusion doses. The infusion maintenance dose can result in even more blood loss reduction. We did not measure the intraoperative serum MgSO_4_ levels. Hence, we avoided using the infusion maintenance dose. Moreover, we used a sample size of 50, which is relatively small.

## Conclusions

The results of this study indicate that a single intravenous bolus of MgSO_4_ effectively reduces intraoperative blood loss, leading to a more precise surgical field during FESS. We have also concluded that MgSO_4_ can help alleviate stress during laryngoscopy and endotracheal intubation. There was no significant difference in intraoperative fentanyl requirement or extubation time between the groups. These findings suggest the potential benefits of using MgSO_4_ in FESS surgery. Further research could explore the possibility of continuous intraoperative infusion of MgSO_4_ and its use in other surgical procedures.
